# Chlorophyll-Loaded Castor Oil Nanoemulsions Exhibit Photodynamic Therapy Efficacy Against B16-F10 Melanoma with Low Cytotoxicity Toward HaCaT Keratinocytes

**DOI:** 10.3390/ph19070974

**Published:** 2026-06-23

**Authors:** Joabe Lima Araújo, Alexandre Silva Santos, Vitória Regina Miranda Carvalho Silva, Lucas Carvalho dos Santos, André de Lima e Silva Mariano, Isadora Florêncio, Sônia Nair Báo, Sebastião William da Silva, Paulo Eduardo N. Souza, Ricardo Bentes Azevedo, Luís Alexandre Muehlmann

**Affiliations:** 1 Department of Genetics and Morphology, Institute of Biological Sciences, Darcy Ribeiro University Campus, University of Brasília, Brasília 70910-900, Brazil; vitoria.rmcs@gmail.com (V.R.M.C.S.); razevedo@unb.br (R.B.A.); 2 Optical Spectroscopy Laboratory, Institute of Physics, Darcy Ribeiro University Campus, University of Brasília, Brasília 70910-900, Brazil; alexandre.silva@unb.br (A.S.S.); demariano95@gmail.com (A.d.L.e.S.M.); swsilva@unb.br (S.W.d.S.); psouza@unb.br (P.E.N.S.); 3 Institute of Chemistry, Darcy Ribeiro University Campus, University of Brasília, Brasília 70910-900, Brazil; lucantos4@gmail.com; 4 Cellular Biology Department, Institute of Biological Sciences, Darcy Ribeiro University Campus, University of Brasília, Brasília 70910-900, Brazil; isadoraflorenciofs@gmail.com (I.F.); snbao@unb.br (S.N.B.); 5 Faculty of Health Sciences and Technologies, University of Brasilia, Brasília 70910-900, Brazil

**Keywords:** photodynamic therapy, nanoemulsions, chlorophyll, human keratinocytes, melanoma, skin cancer

## Abstract

**Background**: Photodynamic therapy (PDT) is a promising minimally invasive approach for melanoma; however, many photosensitizers lose activity in aqueous media due to aggregation-induced quenching effects. **Objectives**: The aim of this study was to develop and characterize castor oil–based nanoemulsions containing chlorophyll (NFs-Chl) and to evaluate their in vitro photodynamic potential against melanoma cells (B16-F10), as well as their selectivity compared with human keratinocytes (HaCaT). **Methods**: NFs-Chl were prepared by spontaneous emulsification. Physicochemical characterization was carried out using dynamic light scattering (DLS), UV–Vis spectroscopy, FTIR, and Raman spectroscopy. In vitro assays included MTT for cell viability (IC_50_ determination), real-time cell proliferation (RealTime-Glo™), and cell migration analysis (scratch assay). All photodynamic treatments were performed under irradiation at 660 nm. **Results**: NFs-Chl exhibited homogeneous nanometric sizes (≈24–31 nm) and a low polydispersity index (≈0.25–0.40), indicating a narrow size distribution. UV–Vis spectra confirmed the preservation of the characteristic absorption peaks of chlorophyll after encapsulation. In B16-F10 cells, NFs-Chl associated with PDT significantly reduced cell viability and metabolic activity over 48 h. Furthermore, NFs-Chl inhibited the migratory capacity of B16-F10 cancer cells. Cell migration assays revealed a clear inhibition of B16-F10 cell migration following treatment with NFs-Chl + PDT. **Conclusions**: Encapsulation of chlorophyll into castor oil nanoemulsions protected the photosensitizer, improved its cellular delivery, and enhanced its photodynamic cytotoxic effect against melanoma cells, while relatively preserving normal keratinocytes in vitro.

## 1. Introduction

Cancer remains one of the leading causes of morbidity and mortality worldwide. Among the different cancer types, melanoma represents a major public health concern because of its aggressive behavior, high metastatic potential, and increasing incidence rates worldwide [1,2]. Studies by Flatz and Reitmajer [2] report that melanoma is among the most prevalent cancers in fair-skinned populations, particularly cutaneous melanoma, with higher incidence rates observed in Europe, Australia, and the United States. The study further indicates that, in 2020, there were approximately 325,000 new cases and 57,000 deaths attributable to cutaneous melanoma worldwide. Moreover, current trends suggest a continued increase in both diagnoses and mortality, with the global burden of melanoma projected to reach approximately 510,000 new cases (an increase of about 50%) and 96,000 deaths (an increase of about 68%) by 2040, representing a significant challenge for healthcare systems worldwide.

Melanoma is the most aggressive form of skin cancer, arising from the malignant transformation of melanocytes, the cells responsible for melanin production [3]. Clinically, melanoma may present as cutaneous, ocular, or mucosal melanoma; however, cutaneous melanoma is the most prevalent subtype worldwide, accounting for approximately 91.3% of newly diagnosed melanoma cases [3].

The clinical diagnosis of cutaneous melanoma may vary according to the disease subtype. The most frequently observed forms include superficial spreading melanoma, nodular melanoma, lentigo maligna melanoma, and acral lentiginous melanoma. Among these, superficial spreading melanoma is the most prevalent subtype, accounting for approximately 70–75% of melanoma diagnoses worldwide [4].

An accurate and early diagnosis is essential to initiate appropriate treatment. Therapeutic management may involve invasive approaches, such as surgical excision of the tumor, followed by chemotherapy and/or radiotherapy, which constitute conventional treatment modalities and significantly increase the chances of cure [5]. In addition, melanoma may also be treated using immunotherapy or immunochemotherapy, which involve the intravenous administration of therapeutic agents designed to stimulate the patient’s immune system to recognize and inhibit tumor cells [6].

However, in more aggressive cases, such as metastatic melanoma, currently available therapeutic strategies present important limitations, particularly with respect to therapeutic resistance. These limitations persist even with newer treatment approaches, including immunochemotherapy, biochemotherapy, and melanoma stem cell–targeted strategies. Moreover, the occurrence of significant adverse effects may compromise patient adherence to treatment, further complicating clinical management [7].

Therefore, the development of therapeutic alternatives with greater efficacy and fewer adverse effects for the treatment of metastatic melanoma remains essential for advancing melanoma management. In this context, photodynamic therapy (PDT) emerges as a promising therapeutic strategy, as it offers several advantages. These include high tissue and site specificity, since photosensitization occurs only in regions where the photosensitizer is administered; minimal invasiveness, as it does not require surgical intervention and causes reduced damage to surrounding tissues, thereby improving patient adherence to treatment; low systemic toxicity; and the possibility of repeated treatment sessions due to its generally well-tolerated and minimally painful nature.

Furthermore, PDT may reduce the likelihood of therapeutic resistance in cancer cells because its mechanism of action involves the generation of reactive oxygen species (ROS), which induce oxidative damage to cellular components, including DNA, proteins, and membranes. Consequently, this mechanism confers a lower probability of resistance development compared with conventional therapies that rely primarily on cytotoxic compounds [8].

However, PDT is only effective when a photosensitizer generates ROS upon photoexcitation. This process occurs when the photosensitizer reaches an excited state after exposure to monochromatic light at a specific wavelength. In the presence of molecular oxygen (O_2_), the excited photosensitizer transfers energy to oxygen, leading to the formation of ROS, particularly singlet oxygen (^1^O_2_). These reactive species induce oxidative damage to cellular components, including DNA, ultimately triggering cancer cell death through apoptosis or necrosis [9].

Therefore, the selection of an appropriate photosensitizer is critical for the effectiveness of PDT. In this context, chlorophyll, a naturally occurring photosensitizer, represents a promising alternative when combined with PDT for the treatment of melanoma. This compound exhibits several favorable photophysical properties, including low toxicity, high triplet-state yield, efficient energy transfer to molecular oxygen, and a high quantum yield for the generation of singlet oxygen (^1^O_2_), as highlighted by Semeraro et al. [10]. Moreover, chlorophyll is abundant in nature and can be readily obtained from various biological sources, including algae, plant leaves, and cyanobacteria.

However, several limitations hinder the clinical application of chlorophyll as a photosensitizer in PDT. Chlorophyll is a highly hydrophobic molecule that is prone to self-aggregation in aqueous solutions, a phenomenon that significantly reduces its fluorescence quantum yield, triplet-state formation, and ROS generation capacity, ultimately impairing its photosensitizing activity [11]. Considering that drug candidates should preferably be formulated in aqueous media to ensure greater biocompatibility and improved bioavailability [12], the use of chlorophyll as an isolated compound becomes clinically impractical.

To overcome these limitations, nanobiotechnology, particularly through the use of nanoemulsions, represents a promising strategy due to its capacity to encapsulate hydrophobic molecules and confer advantageous properties, such as improved colloidal and thermodynamic stability, controlled or sustained drug release, and enhanced biocompatibility and bioavailability [12]. Thus, encapsulating chlorophyll within nanoemulsions for application in PDT against metastatic or non-metastatic melanoma may provide a viable alternative therapeutic approach for this malignancy.

However, limited information is available regarding chlorophyll-loaded castor oil nanoemulsions capable of simultaneously preserving the photophysical properties of chlorophyll, maintaining long-term physicochemical stability, and promoting photodynamic activity against melanoma cells. Furthermore, studies investigating the relationship between chlorophyll encapsulation, ROS generation, and photodynamic biological activity in melanoma models remain scarce.

Therefore, the novelty of the present study lies not only in the development of a chlorophyll-loaded castor oil nanoemulsion but also in the comprehensive investigation of the relationship between nanoformulation physicochemical stability, ROS generation capacity, and photodynamic biological performance against melanoma cells. To the best of our knowledge, studies integrating long-term stability evaluation (365 days), physicochemical characterization, ROS production, and multiple in vitro biological endpoints within a single chlorophyll-based nanoemulsion platform remain limited in the current literature. Accordingly, the objective of the present study was to develop and characterize a nanoformulation containing chlorophyll as the photosensitizing agent associated with PDT and to evaluate its activity against murine melanoma cancer cells (B16-F10).

## 2. Results and Discussion

### 2.1. Chlorophyll Nanoformulations

To obtain the optimal chlorophyll nanoformulation (NFs-Chl), formulations were optimized to achieve the most favorable physicochemical properties, as determined by dynamic light scattering (DLS) analysis using a ZetaSizer Nano ZS^®^ (Malvern Instruments, Malvern, UK), as well as the highest photodynamic activity.

**Figure 1:**
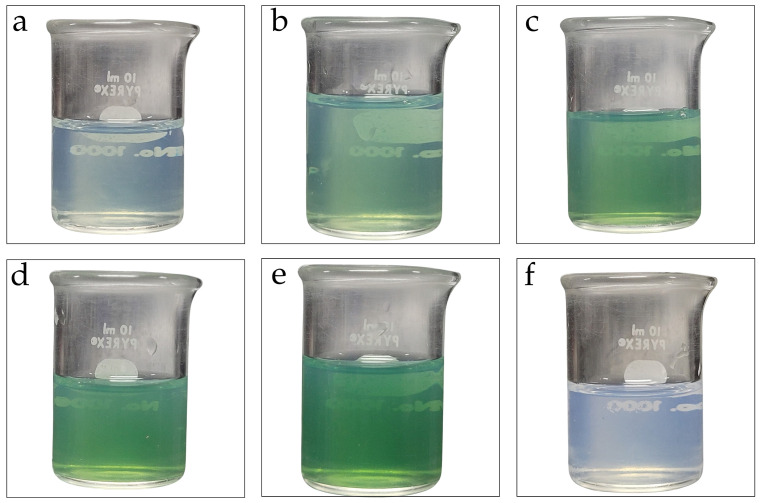
Chlorophyll nanoformulations (NFs-Chl) prepared at different concentrations and a chlorophyll-free nanoemulsion. Note: (**a**) NFs-Chl containing 168 µM chlorophyll; (**b**) NFs-Chl containing 420 µM chlorophyll; (**c**) NFs-Chl containing 840.5 µM chlorophyll; (**d**) NFs-Chl containing 1260 µM chlorophyll; (**e**) NFs-Chl containing 1680 µM chlorophyll; and (**f**) chlorophyll-free nanoemulsion.

Accordingly, a screening of NFs-Chl at different concentrations was performed in order to identify a nanoformulation capable of efficiently encapsulating chlorophyll while preserving its photosensitizing capacity to generate reactive oxygen species (ROS) when excited by light at a wavelength of 660 nm. As a result, different NFs-Chl formulations at varying concentrations were obtained, all presenting a translucent appearance, as illustrated in Figure 1.

Following the preparation of translucent NFs-Chl formulations, their physicochemical properties were characterized, including hydrodynamic diameter (HD) and polydispersity index (PdI), using dynamic light scattering (DLS). The zeta potential (ZP) of the nanodroplets was determined by electrophoretic mobility measurements using laser Doppler electrophoresis (LDE), performed with the same instrument (ZetaSizer Nano ZS^®^) on the day of formulation. The results indicate that the castor oil nanoemulsions successfully encapsulated chlorophyll and exhibited favorable physicochemical characteristics. Specifically, the PdI values obtained for the NFs-Chl ranged from 0.056 to 0.126, while the hydrodynamic diameters varied between 24.14 and 30.25 nm. In addition, it was observed that increasing chlorophyll concentrations led to a corresponding increase in the hydrodynamic diameter of the nanodroplets (Table 1).

These results indicate that the formulated chlorophyll nanoformulations (NFs-Chl) exhibited a uniform particle size distribution pattern. Such a characteristic is advantageous for clinical applications, as it enhances the predictability of the physicochemical properties of the nanoformulations, thereby improving bioavailability and facilitating targeted delivery to specific sites [13]. The results also revealed a progressive increase in nanodroplet size as the chlorophyll concentration increased. This trend is expected, since higher concentrations of the active compound tend to expand the interfacial region of the nanodroplets, resulting in a gradual increase in their hydrodynamic diameter [14].

### 2.2. Optical Characterization by UV–Vis Spectroscopy

**Figure 2:**
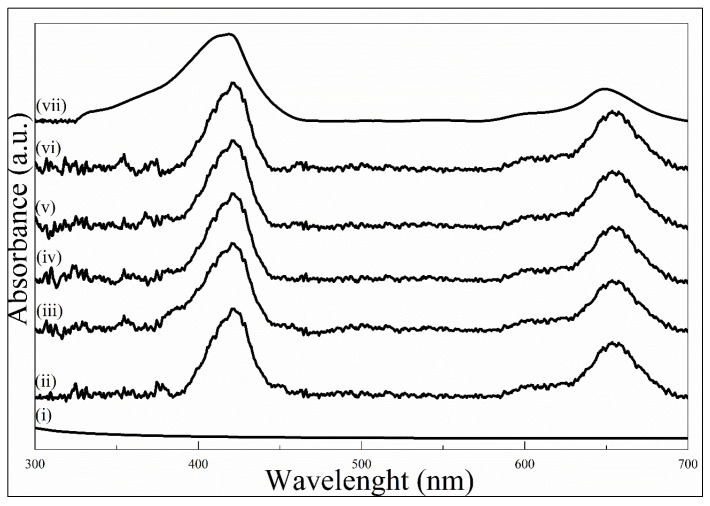
UV–Vis spectra of chlorophyll-free nanoemulsions and NFs-Chl at different concentrations. Note: (i) chlorophyll-free nanoemulsion; (ii) NFs-Chl containing 168 µM chlorophyll; (iii) NFs-Chl containing 420 µM chlorophyll; (iv) NFs-Chl containing 840 µM chlorophyll; (v) NFs-Chl containing 1260 µM chlorophyll; (vi) NFs-Chl containing 1680 µM chlorophyll; and (vii) commercial chlorophyll-a solution in ethanol used as the standard.

To elucidate whether the photosensitizing capacity of chlorophyll was preserved, UV–Vis spectroscopy analyses were performed in order to identify the characteristic absorption peaks of chlorophyll. In Figure 2, the UV–Vis spectra of NFs-Chl at different concentrations are presented: (i) chlorophyll-free nanoemulsion; (ii) NFs-Chl containing 168 µM chlorophyll; (iii) NFs-Chl containing 420 µM chlorophyll; (iv) NFs-Chl containing 840.5 µM chlorophyll; (v) NFs-Chl containing 1260 µM chlorophyll; (vi) NFs-Chl containing 1680 µM chlorophyll; and (vii) a standard chlorophyll-a solution (Sigma-Aldrich) diluted in ethanol, used as the control.

It can be observed that the chlorophyll-free nanoemulsion did not exhibit absorbance peaks within the scanned wavelength range of 300–700 nm, as reported in the measurements. This is expected because the S/O mixture used in the formulation of the chlorophyll-free nanoemulsions does not possess photosensitizing properties. In contrast, the NFs-Chl at different concentrations displayed two characteristic absorbance peaks: one in the blue spectral region at approximately 420 nm and another in the red region at approximately 655 nm (Figure 2). Similarly, the UV–Vis spectrum of the commercial chlorophyll-a solution also presented two absorbance peaks, one in the blue spectral region at 415 nm and another in the red region at 660 nm (Figure 2).

The absorbance peaks obtained for the NFs-Chl at different concentrations and for the commercial chlorophyll-a solution are consistent with each other and are also in agreement with values reported in the literature. For instance, the studies of Lichtenthaler and Buschmann [15] describe the characteristic absorbance peaks of chlorophyll-a and chlorophyll-b, which exhibit absorption bands in the blue spectral region at approximately 428 nm and 453 nm, respectively, and in the red spectral region at approximately 661 nm and 642 nm, respectively.

These results confirm that the chlorophyll present in the castor oil used in the NFs-Chl of this study is predominantly chlorophyll-a. The small spectral differences observed between the NFs-Chl and the commercial chlorophyll-a solution are likely influenced by the solvent environment, which alters the polarity of the medium and consequently leads to slight shifts in the spectral bands, even when the same molecule is analyzed [15].

### 2.3. Fourier-Transform Infrared Spectroscopy (FTIR)

**Figure 3:**
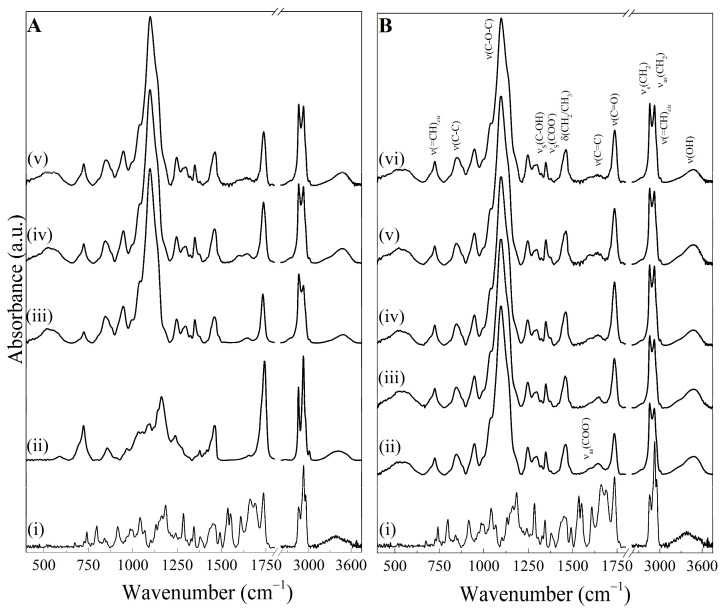
FTIR spectra of the chlorophyll-free nanoemulsion, NFs-Chl, and their individual components. Note: (**A**) Spectra of the isolated constituents: (i) chlorophyll solution; (ii) castor oil; (iii) Cremophor; (iv) chlorophyll-free nanoemulsion; and (v) NFs-Chl containing 1.681 × 10^3^ μM chlorophyll. (**B**) Chlorophyll solution (i); NFs-Chl at concentrations of 168 μM (ii), 420 μM (iii), 840.5 μM (iv), 1260 μM (v), and 1680 μM (vi).

Fourier transform infrared (FTIR) spectroscopy was employed to characterize and identify the functional chemical groups present in NFs-Chl as a function of changes in chlorophyll concentration, as this technique is sensitive to variations in the vibrational properties of interacting molecules. The NFs-Chl formulations and the chlorophyll-free nanoemulsion, as well as the individual components (Cremophor, chlorophyll solution in oil, and castor oil), were analyzed, and the corresponding spectra are presented in Figure 3 along with their respective vibrational assignments.

As shown in Figure 3A, the FTIR spectrum of the NFs-Chl at a chlorophyll concentration of 1680 μM (v) is predominantly characterized by spectral features associated with the chlorophyll-free nanoemulsion (iv), displaying dominant bands attributed to the surfactant (Cremophor) (iii). Under these conditions, the spectral signatures of the isolated components, namely chlorophyll (i) and castor oil (ii), cannot be clearly distinguished.

In Figure 3B, the FTIR spectra of free chlorophyll (i) are presented for comparison with those of the nanoformulations at different concentrations: 168 µM (ii), 420 µM (iii), 840.5 µM (iv), 1260 µM (v), and 1680 µM (vi). When the spectra of the nanoformulations at these concentrations are compared with that of free chlorophyll (i), no distinct spectral features attributable to free chlorophyll are observed, nor are shifts in peak positions or the appearance/disappearance of characteristic bands detected. In this case, the spectra remain similar to that of the chlorophyll-free nanoemulsion, as shown in Figure 3A.

However, this observation does not necessarily indicate the absence of chlorophyll in the nanoformulations. Instead, it suggests that a more sensitive analytical technique is required to confirm that the nanoemulsion contains spectral information corresponding to Cremophor, castor oil, and chlorophyll. In this context, Raman spectroscopy represents a more sensitive alternative for detecting such molecular vibrational signatures.

### 2.4. Raman Spectroscopy Analysis

To achieve a better understanding of potential alterations caused by the presence of chlorophyll and other individual constituents in the spectra obtained for the NFs-Chl, the samples were further analyzed using Raman spectroscopy. Although FTIR is a sensitive technique for identifying functional chemical groups and vibrational stretching modes, in the present study, it did not demonstrate sufficient sensitivity to adequately address the question of interest, particularly with respect to the detection of chlorophyll.

**Figure 4:**
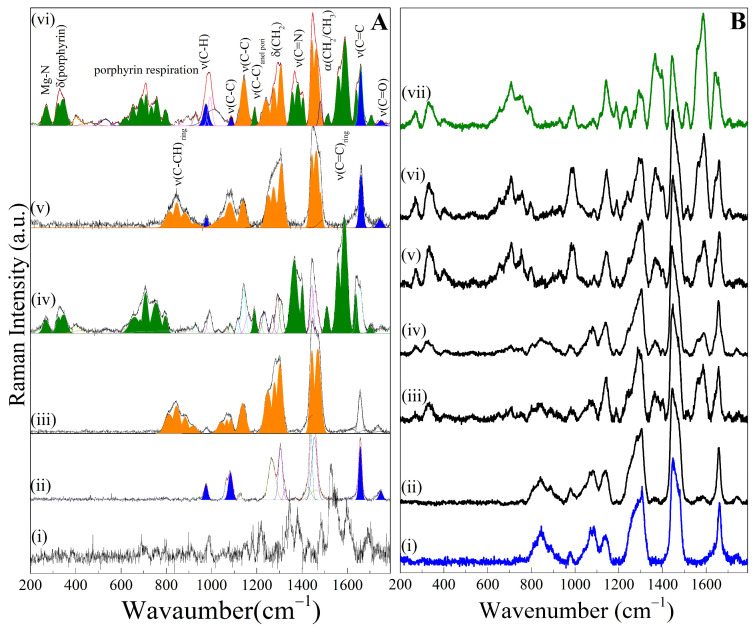
Raman spectra of NFs-Chl and the chlorophyll-free nanoemulsion with their respective individual constituents and at different concentrations. Note: (**A**) NFs-Chl and their individual constituents: (i) chlorophyll solution; (ii) castor oil; (iii) Cremophor; (iv) castor oil + chlorophyll; (v) chlorophyll-free nanoemulsion; and (vi) NFs-Chl containing 1680 μM chlorophyll. (**B**) NFs-Chl at different concentrations of chlorophyll dissolved in ethanol: (i) chlorophyll-free nanoemulsion; (ii) NFs-Chl containing 168 μM chlorophyll; (iii) 420 μM; (iv) 840.5 μM; (v) 1260 μM; (vi) 1680 μM; and (vii) castor oil + chlorophyll. Note: the blue, orange, and green colors in the fittings of (**A**) (vi) are associated with individual contributions of castor oil, Cremophor, castor oil + chlorophyll, respectively. And in (**B**) (i and vii) the blue and green spectra are associated with the chlorophyll-free nanoemulsion and castor oil + chlorophyll samples, to highlight the changes.

In Figure 4A, the Raman spectra of the NFs-Chl (vi) and their respective constituents are presented: (i) chlorophyll solution, (ii) castor oil, (iii) Cremophor, (iv) castor oil + chlorophyll, and (v) chlorophyll-free nanoemulsion, along with their corresponding vibrational assignments. In Figure 4B, for comparison, the resonance Raman spectra of the samples are shown, including the chlorophyll-free nanoemulsion (i), NFs-Chl containing 168 µM (ii), 420 µM (iii), 840.5 µM (iv), 1260 µM (v), 1680 µM (vi), and the chlorophyll solution (vii).

As shown in Figure 4A, the Raman spectrum of the NFs-Chl containing 1680 μM chlorophyll (vi) exhibits spectral features corresponding to castor oil (ii), Cremophor (iii), and castor oil + chlorophyll (iv), highlighted in blue, orange, and green, respectively. These observations indicate that the NFs-Chl spectrum contains characteristic bands associated with both the surfactant and the oil phase, suggesting that the nanoformulation successfully encapsulated chlorophyll. In contrast, the chlorophyll-free nanoemulsion displays only the spectral features associated with castor oil (ii) and Cremophor (iii), as expected, since its composition corresponds to the formulation process used to obtain the blank nanoemulsion.

In Figure 4B, the Raman spectra of NFs-Chl at different chlorophyll concentrations are presented, namely 168 µM (ii), 420 µM (iii), 840.5 µM (iv), 1260 µM (v), and 1680 µM (vi), along with the spectrum of the castor oil + chlorophyll solution (vii). For comparison, the spectra of the chlorophyll-free nanoemulsion (i) and castor oil + chlorophyll (vii) were used as references. It can be observed that decreasing the chlorophyll concentration leads to modifications in some spectral features, particularly when analyzed relative to the highest concentration evaluated. For example, when comparing the spectrum of the castor oil + chlorophyll sample shown in Figure 4B (vii) with that of the NFs-Chl containing 1680 µM chlorophyll in Figure 4A (vi), it is evident that the spectra of the NFs-Chl are predominantly characterized by spectral features similar to those observed for the castor oil + chlorophyll sample in Figure 4B (vii).

These results demonstrate the presence of characteristic spectral features of chlorophyll in the NFs-Chl, such as signals associated with the porphyrin ring, which were observed at nearly all analyzed concentrations (420, 840.5, 1260, and 1680 µM, respectively). The only exception was the NFs-Chl at a concentration of 168 µM, shown in Figure 4B (ii), in which this spectral feature could not be clearly detected. Specifically, the disappearance of bands in the regions of 271 and 327 cm^−1^, typically associated with the porphyrin ring structure of chlorophyll, was observed in this formulation. Additionally, the absence of bands at 1190, 1240, 1365, 1400, 1560, 1590, 1642, and 1706 cm^−1^ further indicates the lack of detectable chlorophyll vibrational signatures. As a result, the Raman spectrum of the NFs-Chl at 168 µM, shown in Figure 4B (ii), exhibits spectral characteristics similar to those of the chlorophyll-free nanoemulsion, shown in Figure 4B (i), when the spectra are compared.

### 2.5. Photodynamic Generation of Reactive Oxygen Species (ROS)

After confirming the physicochemical characteristics of the NFs-Chl by dynamic light scattering (DLS), as well as the molecular vibrations identified by FTIR and their vibrational modes determined by Raman spectroscopy, in addition to the absorption properties characterized by UV–Vis spectroscopy, the ability of the NFs-Chl at different concentrations to induce the generation of ROS was evaluated. ROS generation is a critical factor for the effectiveness of PDT. This analysis aimed to identify the NFs-Chl formulation with the greatest potential for photodynamic applications against melanoma.

To this end, the ability of NFs-Chl to induce ROS generation was evaluated through the degradation of 1,4-diphenyl-2,3-benzofuran (DPBF), a ROS-sensitive probe molecule. Upon interaction with ROS, DPBF undergoes structural rearrangement, forming the colorless compound 1,3-dibenzoylbenzene (DBB), which results in the disappearance of the characteristic absorption band at 410 nm [16].

**Figure 5:**
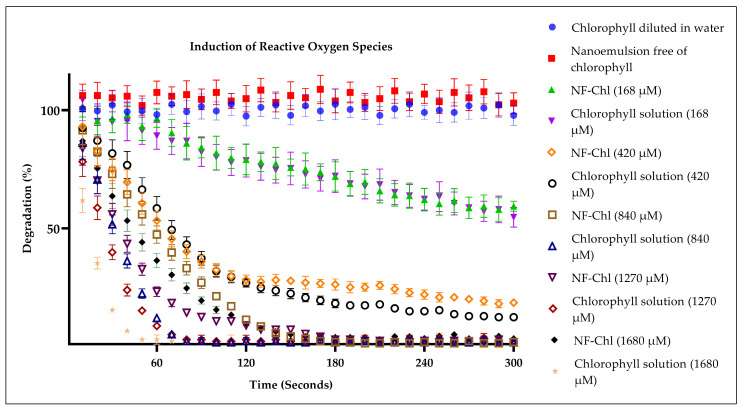
Degradation of 1,4-diphenyl-2,3-benzofuran upon exposure to reactive oxygen species. Note: NFs-Chl, chlorophyll nanoformulation; µM, micromolar.

It can be observed that when the DPBF solution containing chlorophyll samples dispersed in water (H_2_O) and the chlorophyll-free nanoemulsion was exposed to irradiation at a wavelength of 660 nm, the characteristic absorbance peak of DPBF at 410 nm remained unchanged. This result indicates that the chlorophyll samples in H_2_O and the chlorophyll-free nanoemulsion did not induce the generation of ROS, and, therefore, no molecular rearrangement of DPBF into 1,3-dibenzoylbenzene (DBB) occurred, as shown in Figure 5.

In contrast, an opposite effect was observed for the NFs-Chl at different concentrations, as well as for the chlorophyll solution diluted in absolute ethanol, where a progressive decrease in the DPBF absorbance peak was detected as the irradiation time approached 300 s. This decrease indicates DPBF degradation due to its reaction with ROS, leading to molecular rearrangement and the formation of the colorless compound DBB. Consequently, the characteristic DPBF absorbance peak at 410 nm was completely suppressed when exposed to NFs-Chl containing 840 µM and 1260 µM chlorophyll.

These results demonstrate that the NFs-Chl overcome the limitations associated with chlorophyll dilution and aggregation in aqueous suspensions. When chlorophyll is not encapsulated in nanoemulsions, its photodynamic activity is inhibited due to its hydrophobic nature, which promotes molecular aggregation and consequently leads to quenching of its photosensitizing activity [17].

Another observation from this analysis was the limited ROS generation capacity of NFs-Chl at the lowest chlorophyll concentration (168 µM), as shown in Figure 5. The characteristic DPBF absorbance peak at 410 nm remained detectable even after 300 s of LED irradiation in the presence of NFs-Chl containing 168 µM chlorophyll. This result indicates that this chlorophyll concentration in the nanoformulation is insufficient to generate ROS in amounts capable of completely degrading DPBF. In this context, correlating this analysis with the results obtained by Raman spectroscopy reveals agreement between the findings. Specifically, NFs-Chl at a concentration of 168 µM did not induce ROS at levels sufficient to promote complete DPBF degradation, as previously discussed. Moreover, at this same concentration (168 µM), the spectral characteristics of the NFs-Chl and the chlorophyll-free nanoemulsion were essentially indistinguishable, with no significant spectral differences observed in the Raman spectra.

Furthermore, for NFs-Chl at the highest chlorophyll concentration (1680 µM), a residual DPBF absorbance signal at 410 nm was still observed, although at low intensity. This phenomenon may occur because the nanodroplets reach their maximum chlorophyll loading capacity, once this limit is exceeded, chlorophyll molecules tend to aggregate, thereby suppressing their photosensitizing activity. This effect has been widely reported in the literature, where nanoformulations that reach their maximum encapsulation capacity often exhibit a reduction in the functional properties of the encapsulated active compound [18,19]. Therefore, these results indicate that the optimal chlorophyll concentration to be encapsulated in the nanoemulsion system should be below 1680 µM. In this context, NFs-Chl formulations exhibiting minimal ROS-inducing activity at 168 µM, as well as formulations at concentrations below 1680 µM (for example, 1500 µM), should be considered suitable for subsequent in vitro biological assays. This is because the concentration range between 168 and 1500 µM encompasses the effective photodynamic interval identified in the ROS-induction experiments, particularly the range between 840 and 1260 µM, in which optimal ROS generation was observed.

Therefore, the NFs-Chl stock formulation initially prepared at 1680 µM was diluted to 1500 µM, and this concentration was subsequently selected to standardize the remaining physicochemical characterization studies and in vitro biological assays, being defined as the standard NFs-Chl formulation. From this standard formulation (1500 µM), different dilution ranges could be obtained within the effective concentration interval identified in the ROS-induction analyses, particularly for the biological assays involving in vitro PDT against melanoma.

Importantly, the 1500 µM formulation was employed as the standard nanoformulation for subsequent biological investigations and not as a single fixed treatment concentration. For the MTT assays, serial dilutions ranging from 93.75 to 1500 µM were prepared from this standard formulation to establish the dose–response profile and determine the IC_50_ value. Subsequently, the IC_50_-derived concentration was used as a biologically relevant reference for complementary proliferation and migration assays.

### 2.6. Optical Stability by UV–Vis Spectroscopy and Fluorescence Emission

**Figure 6:**
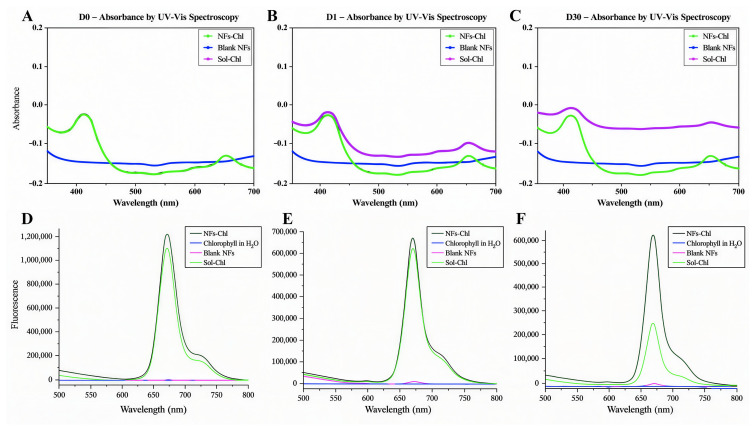
Absorbance and fluorescence spectra of NFs-Chl and chlorophyll solution. (**A**) Absorbance spectra obtained on day 0 (D0); (**B**) absorbance spectra obtained on day 1 (D1); (**C**) absorbance spectra obtained on day 30 (D30); (**D**) fluorescence emission spectra obtained on D0; (**E**) fluorescence emission spectra obtained on D1; and (**F**) fluorescence emission spectra obtained on D30. Note: NFs-Chl, chlorophyll nanoformulation; Blank NFs, chlorophyll-free nanoemulsion; Sol-Chl, chlorophyll solution diluted in ethanol; Chlorophyll in H_2_O, chlorophyll diluted in water; D0, D1, and D30 correspond to the days on which the measurements were obtained.

To evaluate the efficiency of the nanoformulations in preserving the optical properties of chlorophyll, stability analyses of these properties were performed over a period of 30 days using UV–Vis spectroscopy and fluorescence spectroscopy, as shown in Figure 6.

It can be observed that on the day of preparation of the NFs-Chl (1500 µM) (D0), the characteristic chlorophyll absorbance wavelengths at 420 and 665 nm were detected for both the chlorophyll solution diluted in ethanol and the NFs-Chl, as shown in Figure 6A. Subsequently, 24 h after the first analysis (D1), a second evaluation of the NFs-Chl and the chlorophyll solution was performed. The results indicated that the optical absorbance and fluorescence properties remained stable for the NFs-Chl (Figure 6B,E).

In contrast, for the chlorophyll solution, a decrease in the intensity of the absorbance peaks at 420 and 665 nm was observed (Figure 6C), which had previously overlapped with the absorbance peaks of the NFs-Chl (Figure 6A). This finding indicates a loss of optical properties due to chlorophyll degradation after 24 h following solution preparation. This effect is likely associated with variations in pH, temperature, and other environmental factors that contribute to chlorophyll degradation, as this molecule is known to be highly unstable [20].

After 30 days following the preparation of the NFs-Chl and the chlorophyll solution, a drastic reduction in the intensity of the absorbance and fluorescence peaks of the chlorophyll solution was observed, whereas the peaks associated with the NFs-Chl remained stable (Figure 6C,F). These findings demonstrate that the NFs-Chl effectively preserve chlorophyll stability over time, maintaining its photosensitizing properties. Moreover, this result indicates that the nanoformulations are suitable nanocarriers for chlorophyll, largely overcoming limitations related to hydrophobicity and molecular instability.

These findings are particularly relevant considering the well-known limitations of chlorophyll, including its susceptibility to degradation and aggregation in aqueous environments [11]. In contrast, NFs-Chl preserved the characteristic UV–Vis absorption bands and fluorescence emission profile of chlorophyll throughout the evaluation period, indicating improved photophysical stability. Importantly, the preservation of photoactivity was reflected in the subsequent biological assays, which demonstrated reductions in melanoma cell viability, proliferation, and migration following in vitro PDT treatment. Collectively, these results indicate that nanoencapsulation successfully addressed the technical limitations associated with free-chlorophyll instability and loss of functional properties in aqueous media, representing a promising strategy for future in vivo investigations and the development of chlorophyll-based photodynamic therapies against melanoma.

### 2.7. Morphological Analysis by Transmission Electron Microscopy (TEM)

**Figure 7:**
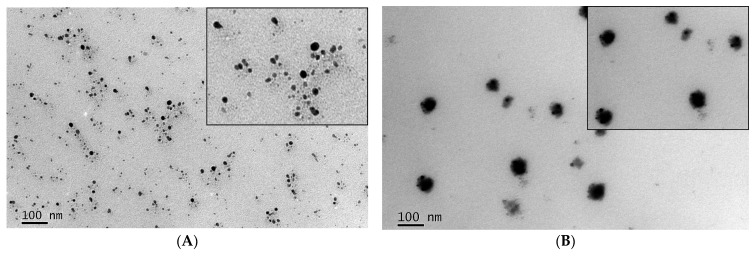
Morphological characteristics: (**A**) chlorophyll-free nanoemulsion; (**B**) chlorophyll-containing nanoformulations.

The NFs-Chl (1500 µM) were analyzed for their morphological characteristics using transmission electron microscopy (TEM). As shown in Figure 7A, the chlorophyll-free nanoemulsion exhibits spheroidal structures with high electron contrast in the central region, indicating a high electron density at the core of the nanodroplets. This feature is likely influenced by the presence of Cremophor, an effect previously reported in studies involving nanoemulsions formulated with this surfactant [21,22]. In contrast, the NFs-Chl display a distinct morphological appearance compared with the chlorophyll-free nanoemulsions, exhibiting more irregular edges and spheroidal shapes with an opaque appearance under TEM, as illustrated in Figure 7B.

**Figure 8:**
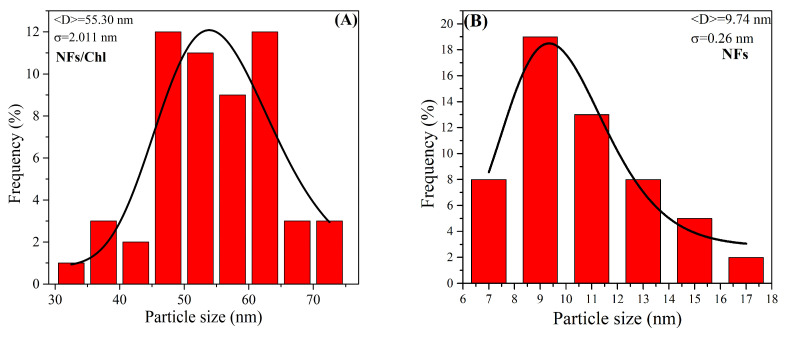
Histograms obtained from TEM images and their corresponding log-normal function fits for NFs-Chl (**A**) and chlorophyll-free NFs (**B**).

The TEM micrographs (Figure 7A,B) enabled morphometric analysis and the construction of size distribution histograms for the nanodroplets of the chlorophyll-free nanoformulations (NFs) and chlorophyll-loaded nanoformulations (NFs-Chl), as shown in Figure 8A,B. The obtained histograms and their corresponding fits using a log-normal function reveal particle size distributions typical of systems formed through nucleation and growth processes governed by interfacial phenomena [23].

The chlorophyll-free NFs exhibited an average diameter of approximately 9.7 ± 0.26 nm, indicating small and well-defined nanodroplets with a narrow size dispersion. In contrast, the NFs/Chl presented a significantly larger average diameter, approximately 55.3 ± 2.0 nm, while still maintaining a particle distribution with good morphological homogeneity, as observed in TEM micrographs.

This size difference confirms that the systems correspond to distinct nanoformulations and that the incorporation or encapsulation of chlorophyll altered the balance of interfacial forces during the emulsification process. This effect may reduce the packing efficiency of the surfactant (Cremophor ELP^®^) around the castor oil droplets, thereby favoring the formation of larger nanodroplets. In addition, hydrophobic interactions between chlorophyll molecules may contribute to partial coalescence or stabilization of larger structures [12].

It should be emphasized that the apparent discrepancy between the diameters obtained by TEM and DLS should not be interpreted as distinct or contradictory differences. DLS reports the hydrodynamic diameter of nanodroplets dispersed in liquid, whereas TEM provides a morphological measurement of dehydrated particles. Since DLS includes the solvation layer and is based on Brownian diffusion, while TEM is subject to drying-related artifacts during grid preparation, the two methods probe different physical dimensions of the same colloidal system. Consequently, the larger TEM-derived size observed for NFs-Chl is consistent with the expected behavior of soft nanoemulsion systems and should be interpreted as complementary to the DLS results.

### 2.8. Colloidal Stability of Chlorophyll Nanoformulations over 365 Days

**Figure 9:**
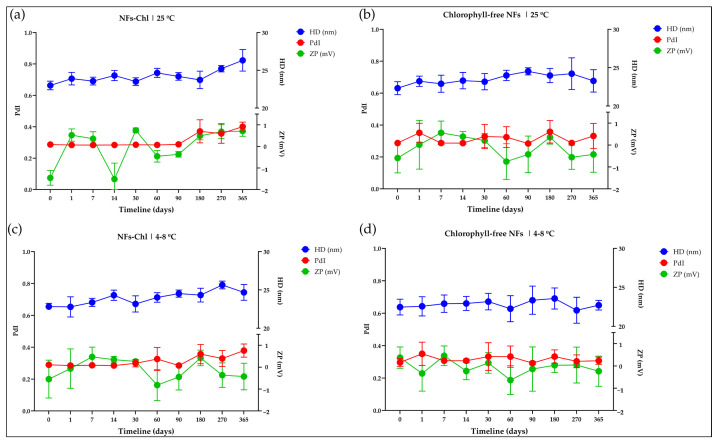
Stability of NFs-Chl (1500 µM) over 365 days determined by DLS under different storage temperatures: (**a**) NFs-Chl stored at 25 °C; (**b**) chlorophyll-free NFs stored at 25 °C; (**c**) NFs-Chl stored at 4–8 °C; and (**d**) chlorophyll-free NFs stored at 4–8 °C. Note: DLS, dynamic light scattering; NFs-Chl, chlorophyll-loaded nanoformulations; HD, hydrodynamic diameter; PdI, polydispersity index; ZP, zeta potential; °C, degrees Celsius; nm, nanometer; mV, millivolt.

The NFs-Chl and the chlorophyll-free NFs were evaluated for their colloidal physicochemical characteristics and nanodroplet stability over a period of 365 days under two storage conditions: room temperature (25 °C) and refrigerated conditions (4–8 °C), as shown in Figure 9.

As shown in Figure 9a, the mean hydrodynamic diameter (HD, blue curves) of the NFs-Chl remained stable over the 365-day period, within an approximate range of ~22–28 nm, with only minor variations (±~2–3 nm) and a slight increase observed near the end of the monitoring period. The polydispersity index (PdI, red) was predominantly within the range of ~0.25 to 0.40, with occasional transient peaks indicating a temporary increase in heterogeneity at certain time points (notably between 180 and 365 days). The zeta potential (ZP, green) fluctuated around neutrality (approximately −1 to +1 mV) throughout the entire period, exhibiting minor oscillations but without a clear trend toward strongly positive or negative values.

Similarly, for the chlorophyll-free NFs shown in Figure 9b, the mean hydrodynamic diameter (HD) remained consistent (~22–26 nm) throughout the one-year period, with no abrupt changes observed. The PdI also remained within a moderate range (~0.25–0.40), displaying only minor fluctuations over time. The ZP exhibited slight oscillations around neutrality, with larger experimental errors at certain time points, but without any clear increase in magnitude that would indicate changes in electrostatic stability.

In formulations stored under refrigerated conditions (4–8 °C), the HD trend was comparable to that observed at room temperature. The mean HD values remained around 22–27 nm, showing only minor variability and a moderate peak near the end of the monitoring period, a pattern similar to that observed under storage at 25 °C. The PdI remained within the range of 0.25–0.40, with occasional points indicating increased heterogeneity at intermediate time intervals. The ZP alternated between slightly positive and negative values, remaining close to neutrality (approximately −1 to +1 mV). Overall, no evidence of reduced stability was observed under refrigerated storage compared with storage at 25 °C. In both storage conditions, the formulations behaved similarly when their colloidal physicochemical characteristics were evaluated by the DLS technique.

The ZP values observed in Figure 9a–d fluctuated between −1 mV and +1 mV throughout the 365-day analysis period. Minor fluctuations were observed at specific time points; in some cases, the error bars for ZP were noticeably larger, indicating greater experimental variability at those points. However, no statistically significant differences were observed relative to D0.

The nanodroplet sizes observed in the present study (~22–28 nm), together with PDI values ranging from approximately 0.25 to 0.40, are considered acceptable for topical nanoformulations. Within this size range, skin penetration is expected to occur predominantly through the transfollicular pathway. Furthermore, PDI values below 0.7, combined with the long-term preservation of hydrodynamic diameter and the absence of macroscopic signs of instability, such as phase separation or droplet growth, provide additional evidence supporting the colloidal stability of the NFs-Chl formulations throughout the storage period [24].

When compared with recent literature on nanoformulations, the data presented here are noteworthy in two main aspects. First, the prolonged stability at room temperature was observed without the apparent need for lyophilization or storage at low temperatures. Several studies report limited stability for lipid-based formulations and other nanoparticle systems, often identifying freezing or lyophilization as necessary strategies to ensure long-term stability and preservation of physicochemical properties. For example, the study by Ai et al. [25] reports that lipid nanoparticles (LNPs) and other nanoformulations frequently require refrigerated storage or lyophilization to maintain stability beyond a few months.

The second noteworthy aspect lies in the ability of NFs-Chl to remain stable while exhibiting a ZP of approximately 0 mV, without a significant increase in nanodroplet size. This behavior is generally unexpected from a technical standpoint, since ZP values close to neutrality are typically associated with low electrostatic repulsion and a higher tendency toward particle aggregation. As discussed by Burlec et al. [26], in studies on colloidal stability, more extreme ZP values, for example, >+30 mV or <−30 mV, are commonly correlated with electrostatically stable dispersions, whereas values near zero increase the likelihood of agglomeration unless steric stabilization mechanisms are present. In this context, the long-term preservation (365 days) of HD and PdI observed herein indicates that the stability of the NFs-Chl is not predominantly governed by electrostatic repulsion, but rather by interfacial steric stabilization. This interpretation is consistent with the presence of the nonionic surfactant Cremophor ELP^®^ in the NFs-Chl formulations, which likely formed a protective interfacial layer surrounding the castor oil nanodroplets and prevented droplet coalescence even under near-neutral zeta-potential conditions.

These results demonstrate that NFs-Chl did not undergo aggregation during the 365 days following production, regardless of storage temperature (4–8 °C or 25 °C). This finding indicates that NFs-Chl may be industrially attractive, as they exhibit physicochemical stability, which enhances chlorophyll bioavailability and shelf life (under light-protected conditions), while allowing straightforward storage independent of temperature, thereby facilitating transportation and stockpiling. In addition, the low-energy preparation process makes these nanoformulations particularly attractive for scale-up without the need for complex or high-cost equipment, representing a significant industrial advantage [14,27].

A difference in particle size was also observed when comparing measurements obtained by dynamic light scattering (DLS) and transmission electron microscopy (TEM). DLS analysis showed a mean HD of approximately ~22–28 nm for NFs-Chl and ~22–26 nm for the chlorophyll-free NFs. In contrast, particle size analysis by TEM, based on histograms fitted with a log-normal distribution, revealed an average size of approximately ~55 nm for NFs-Chl and ~10 nm for the chlorophyll-free NFs.

This discrepancy is expected and widely documented in the state of the art, as the two techniques measure different physical properties. In this specific case, for NFs-Chl, the presence of chlorophyll may alter the refractive index and optical density of the sample, thereby modifying the scattering response in DLS and influencing the apparent HD [28]. Furthermore, the presence of amphiphilic molecules with predominantly hydrophobic and pigmented characteristics may alter the local viscosity and the hydrodynamic layer surrounding the particles, leading to additional differences between the “optical/hydrodynamic” size obtained by DLS and the “geometric” size determined by TEM.

### 2.9. Cell Viability Assays by MTT

**Figure 10:**
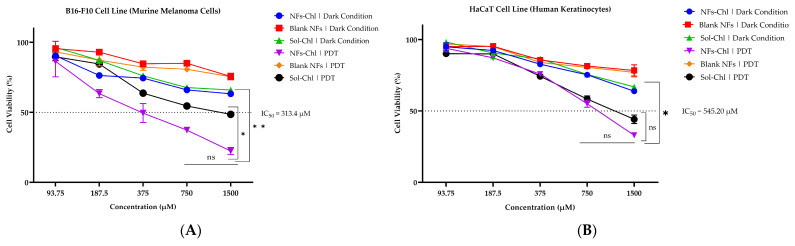
Cell viability analyzed by MTT assay: (**A**) murine melanoma B16-F10 cells treated with NFs-Chl, blank NFs, and Sol-Chl under dark and PDT conditions; and (**B**) human keratinocyte HaCaT cells treated with NFs-Chl, blank NFs, and Sol-Chl under dark and PDT conditions. Note: NFs-Chl, chlorophyll nanoformulations; Blank NFs, chlorophyll-free nanoemulsion; Sol-Chl, chlorophyll solution diluted in ethanol; Control, DMEM; %, percentage; PDT, photodynamic therapy; µM, micromolar. Statistical significance: * *p* < 0.05; ** *p* < 0.01; ns, not significant.

The NFs-Chl were evaluated for their photodynamic activity against murine melanoma cells of the B16-F10 cell line. For this assay, the MTT method was employed [29], through which melanoma cancer cell viability was determined after treatment with in vitro PDT using NFs-Chl at different concentrations, as shown in Figure 10A.

As shown in Figure 10A, the results for PDT associated with NFs-Chl (purple curve in Figure 10A) at different concentrations reveal a pronounced reduction in melanoma cell viability as a function of dose/concentration, resulting in an approximate IC_50_ of 313.4 µM (dashed line). The IC_50_ value was calculated from the complete dose–response curve generated using serial dilutions of the 1500 µM standard nanoformulation. Therefore, the IC_50_ does not correspond to the standard concentration itself, but rather to the mathematically estimated concentration required to reduce cell viability by 50% relative to the control group. It is also noteworthy that the same NFs-Chl under dark conditions maintained high cell viability, remaining above 65% even at the highest concentration of 1500 µM, and exceeding 80% at the lowest concentration of 93.75 µM (Figure 10A).

Statistical analysis by ANOVA demonstrated that NFs-Chl subjected to PDT induced a significant concentration-dependent reduction in B16-F10 cell viability. No statistically significant difference (ns) was observed across the 750–1500 µM range in the B16-F10 cells exposed to NFs-Chl under LED irradiation, indicating a phototoxic response plateau within this interval and suggesting that 750 µM is already sufficient to elicit a biological effect close to the maximum detectable response in the MTT assay. In addition, B16-F10 cells exposed to NFs-Chl at 1500 µM under PDT exhibited significantly lower viability than those maintained under dark conditions (*), indicating that the reduction in cell viability is associated with the presence of the photosensitizing agent and its light activation, rather than with the nanocarrier system alone.

These findings are consistent with the ROS-generation results presented in Section 2.5, which showed that the 840 and 1260 µM formulations generated sufficient ROS to degrade DPBF. Overall, the 750–1500 µM range encompasses the most effective concentrations for ROS induction and for promoting cell death in murine B16-F10 melanoma cells.

These findings demonstrate the low intrinsic cytotoxicity of NFs-Chl in the absence of light activation, a phenomenon that is expected and widely reported in the literature. Effective photosensitizers typically exhibit low cytotoxicity under dark conditions while displaying high cytotoxicity when activated by light at a specific wavelength [30,31].

It was also observed that Sol-Chl at different concentrations, when associated with PDT, reduced the viability of melanoma cells, although with lower efficacy compared with NFs-Chl combined with PDT. The photodynamic activity of Sol-Chl can be attributed to the amphiphilic nature of chlorophyll, which contains a polar porphyrin ring (“head,” with Mg^2+^ coordinated to nitrogen atoms) and a long lipophilic phytol chain. This structure confers both polar and apolar characteristics to the molecule.

Ethanol, a protic polar solvent with partial apolar character, possesses a polar –OH group capable of interacting with the porphyrin ring and an ethyl chain that can interact with the lipophilic phytol moiety of chlorophyll. As a result, ethanol can solvate both the “head” and the hydrophobic tail of the chlorophyll molecule, allowing it to remain soluble in the solvent system [32].

However, chlorophyll is an extremely sensitive molecule that readily undergoes degradation when exposed to organic systems, variations in pH, temperatures above 4–8 °C, oxygen, and light [33]. This instability explains the lower cytotoxic activity of Sol-Chl against melanoma cells when compared with the cytotoxic efficacy of NFs-Chl at the same concentrations. These findings suggest that chlorophyll encapsulation likely enhances intracellular delivery while protecting the photosensitizer from degradation and/or promoting greater generation of reactive oxygen species at the target site when activated by light at a specific wavelength (660 nm).

Taken together, these results indicate that the effectiveness of melanoma cell inhibition is directly associated with the photodynamic action of NFs-Chl when activated by light at a specific wavelength (660 nm). This phenomenon can be clearly illustrated by comparing the cytotoxic effects of NFs-Chl and Sol-Chl under dark conditions, where both treatments induce minimal cell death and consequently maintain high melanoma cell viability (Figure 10A). In contrast, following irradiation, a substantial reduction in cell viability is observed. This behavior is a classical and expected outcome for PDT.

In Figure 10B, a similar profile can be observed, but it is quantitatively less sensitive to PDT associated with NFs-Chl. In this case, cell viability of healthy human epithelial keratinocytes (HaCaT) remained higher at all concentrations compared with melanoma cells exposed to NFs-Chl combined with PDT. The IC_50_ obtained for the HaCaT cell line exposed to NFs-Chl was approximately ~545.2 µM (Figure 10B), indicating that a substantially higher concentration is required to achieve a 50% reduction in cell viability compared with the B16-F10 cell line exposed to the same NFs-Chl under PDT conditions.

From a statistical standpoint, the results showed no significant difference (ns) across the 750–1500 µM concentration range in HaCat cells exposed to NFs-Chl under LED irradiation, suggesting a plateau in the cytotoxic effect within this interval. In addition, the comparison between NFs-Chl under LED irradiation and the corresponding NFs-Chl under dark conditions, both applied to HaCat cells at 1500 µM, revealed a significant difference (*), confirming that the cytotoxic effect against melanoma cells (previous assays) and human keratinocytes is ROS-dependent.

From a practical perspective, this finding indicates a stronger relative antitumor response in the B16-F10 melanoma cells than cytotoxicity toward healthy human keratinocytes (HaCat), suggesting a moderate selectivity index in favor of tumor cells with reduced damage to healthy cells. Such selectivity is a desirable feature in studies investigating alternative cancer therapies [34]. Therefore, these results allow us to infer that NFs-Chl exhibit an in vitro potential to provide greater therapeutic efficacy against murine melanoma cells (B16-F10) while causing less in vitro damage to epidermal cells (HaCat). This suggests that the use of NFs-Chl may result in fewer side effects for patients undergoing melanoma treatment; however, this hypothesis must be further investigated and validated through additional future in vivo studies.

### 2.10. Real-Time Cell Proliferation Assays

**Figure 11:**
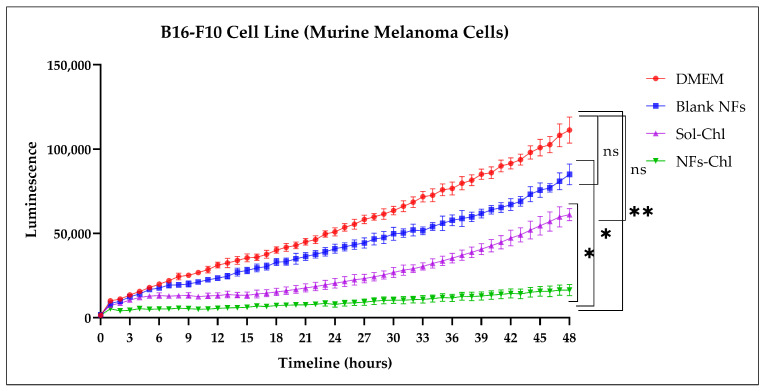
Cell proliferation of melanoma cancer cells (B16-F10 cell line) over 48 h. Note: DMEM, cell culture medium; Blank NFs, chlorophyll-free nanoformulations; Sol-Chl, chlorophyll solution; NFs-Chl, chlorophyll-loaded nanoformulations. Statistical significance: * *p* < 0.05; ** *p* < 0.01; ns, not significant.

Based on the IC_50_ values obtained in the MTT assays, subsequent RealTime-Glo™ proliferation experiments were performed using IC_50_-derived treatment conditions in order to evaluate whether partial viability inhibition was associated with suppression of metabolic/proliferative activity over time. The results of the real-time cell proliferation assays were obtained using the RealTime-Glo™ reagent kit and analyzed with a microplate reader (Varioskan™ LUX, Thermo Scientific, Waltham, MA, USA). In Figure 11, the temporal luminescence curves (0–48 h) corresponding to the proliferation results for the B16-F10 cell line reveal clear differences among the four analyzed samples: (i) control (DMEM); (ii) chlorophyll-free nanoemulsions (blank NFs); (iii) chlorophyll solution in ethanol (Sol-Chl); and (iv) chlorophyll-containing nanoformulations (NFs-Chl).

The control group (DMEM) exhibited the greatest increase in signal over time, indicating high cellular proliferation of B16-F10 melanoma cancer cells and reflecting elevated metabolic activity. Similarly, chlorophyll-free nanoemulsions (blank NFs) also displayed a high metabolic activity signal, indicating substantial cellular proliferation; however, this activity was slightly lower than that observed for the control group. This difference may be attributed to a mild cytotoxic effect of the chlorophyll-free NFs. Nevertheless, this cytotoxic action does not appear sufficient to significantly impair the metabolic capacity of the cells to proliferate over time.

The chlorophyll solution in ethanol (Sol-Chl) associated with PDT exhibited lower luminescence than the chlorophyll-free NFs; however, a relevant level of metabolic activity was still observed, particularly after approximately 15 h of exposure to B16-F10 melanoma cells. This behavior suggests a gradual loss of photodynamic activity over time and possible chlorophyll degradation, which results in increasing metabolic activity after approximately 18 h and continuing to intensify up to 48 h. In contrast, the chlorophyll-containing nanoformulations (NFs-Chl) displayed the lowest metabolic activity signal, remaining close to background levels throughout the entire temporal window (48 h). This finding indicates that the photodynamic activity promoted by NFs-Chl effectively reduced the metabolic activity of B16-F10 melanoma cells, thereby inhibiting cancer cell proliferation in a promising manner.

The enhanced effectiveness of NFs-Chl associated with PDT in suppressing melanoma cell proliferation can be attributed to the advantages provided by nanobiotechnology to the photosensitizer. Nanocarriers can confer greater dispersibility, protection against photosensitizer degradation, and enhanced intracellular uptake [35]. It is important to note that all assays were performed using samples at the previously determined IC_50_ concentration (313.4 µM), obtained from cell viability assays conducted using the MTT method.

These results indicate that the curve generated by NFs-Chl associated with PDT, which shows markedly reduced intensity, reflects a strong impairment of the reductive metabolic activity of B16-F10 melanoma cells at this concentration (IC_50_) and within the evaluated time window (48 h), as measured by the RealTime-Glo™ kit (reduced “viability” biomarker signal). In contrast, the other treatments produced less pronounced or negligible effects when compared with the control group (DMEM).

ANOVA analysis showed that NFs-Chl at the IC_50_ concentration (313.4 µM) maintained B16-F10 cell proliferation at significantly lower levels than the DMEM control group (**), as well as than Sol-Chl (*) and blank NFs (*). In contrast, no statistically significant difference (ns) was observed between DMEM and blank NFs, or between DMEM and Sol-Chl. These findings indicate that the biological effect is specifically associated with the nanoformulated photosensitizer, which is consistent with enhanced cellular interaction and improved intracellular uptake/retention. The significant differences observed between NFs-Chl and both Sol-Chl and blank NFs further confirm that the cytotoxic effect is attributable to the presence of the photosensitizing agent and its light activation, rather than to the nanocarrier system alone.

**Figure 12:**
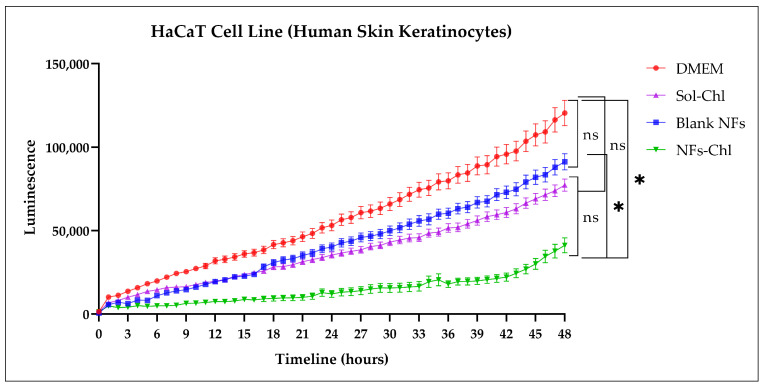
Cell proliferation of human epidermal keratinocytes (HaCaT cell line) over 48 h. Note: DMEM, cell culture medium; Blank NFs, chlorophyll-free nanoformulations; Sol-Chl, chlorophyll solution; NFs-Chl, chlorophyll-loaded nanoformulations. Statistical significance: * *p* < 0.05; ns, not significant.

When analyzing the cell proliferation profile over 48 h for human epidermal keratinocytes of the HaCaT cell line, a progressive increase in luminescence signal is observed over time under all experimental conditions, indicating maintenance of cellular metabolic activity. However, clear differences among the analyzed treatments can be noted, including the control (DMEM), chlorophyll-free nanoformulations (Blank NFs), chlorophyll solution in ethanol (Sol-Chl), and chlorophyll-containing nanoformulations (NFs-Chl), as illustrated in Figure 12.

It can be observed that the control group (DMEM) exhibited the greatest increase in luminescence over the 48 h incubation period, as expected, reflecting the high metabolic activity and normal proliferation of HaCaT keratinocytes under physiological culture conditions (Figure 12). The Blank NFs, for example, also demonstrated a progressive increase in luminescent signal; however, the values were slightly lower than those observed for the control group (DMEM). This suggests that the components of the nanoemulsion may exert a mild cytotoxic effect or induce some degree of cellular stress, as also reported in studies by Kubrak et al. [30]. Nevertheless, this effect was not sufficient to prevent cell proliferation, indicating that the carrier system exhibits relative biocompatibility with skin cells.

NFs-Chl at the IC_50_ concentration maintained B16-F10 cell proliferation at significantly lower levels than the DMEM control group (*) and the blank NFs (*). In contrast, no statistically significant difference (ns) was observed between DMEM and blank NFs, or between DMEM and Sol-Chl. These significant differences observed for NFs-Chl, relative to both DMEM and blank NFs, further confirm that the cytotoxic effect is attributable to the presence of the photosensitizing agent and its light activation, rather than to the nanocarrier system alone.

The results for Sol-Chl exhibited an intermediate behavior, with luminescence values lower than those of the control and NFs-free groups during part of the experiment, while still showing a progressive increase in metabolic activity over time. This pattern suggests that, despite the presence of the photosensitizer, the free form of chlorophyll undergoes degradation with a gradual loss of photodynamic efficiency during the experimental period, allowing the recovery of cellular metabolic activity. This phenomenon is frequently reported in the literature for natural photosensitizers in solution, whose chemical and photodynamic stability tends to be limited when not protected by nanostructured delivery systems [36].

In contrast, the NFs-Chl exhibited the lowest luminescence values throughout the entire analyzed time window. This behavior indicates a more pronounced reduction in the metabolic activity of HaCaT cells when compared with the other treatments, suggesting that the encapsulation of chlorophyll within nanocarriers enhances its photodynamic activity. However, unlike the results observed for the tumor cell line B16-F10 (Figure 11), the inhibitory effect on keratinocytes was moderate, indicating that normal cells retained a certain capacity for proliferation even in the presence of the photosensitizer nanoformulation. This finding suggests a potential degree of biological selectivity of the system, in which tumor cells tend to be more susceptible to oxidative stress generated during photodynamic therapy than normal cells.

### 2.11. Scratch Assay

**Figure 13:**
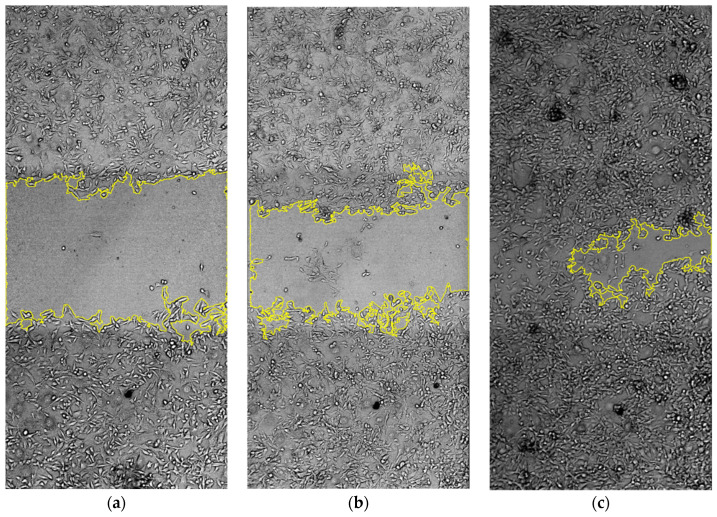
Scratch assay of murine melanoma cells (B16-F10 cell line) exposed to the control group (DMEM). (**a**) B16-F10 at 0 h; (**b**) B16-F10 at 24 h; (**c**) B16-F10 at 48 h.

Likewise, scratch migration assays were conducted using IC_50_-derived treatment conditions to investigate whether the photodynamic effect of NFs-Chl was also associated with reduced collective cell migration relative to the untreated control group. In which, the images obtained using the Invitrogen EVOS FL system (Thermo Fisher Scientific, USA) for B16-F10 cells show the presence of a confluent monolayer at the upper and lower edges, with a well-defined central cell-free region corresponding to the vertical scratch. At 0 h (Figure 13a), the cells located at the upper and lower borders display an elongated (fibroblast-like) morphology with non-condensed nuclei, which is the typical pattern observed for murine melanoma cells of the B16-F10 lineage under active culture conditions. After 24 h (Figure 13b), cells are observed invading the previously cell-free central region, indicating active cell migration. In this stage, elongated cells begin to advance toward the gap, and cells located at the scratch edges exhibit cellular protrusions (filopodia/lamellipodia) adhered to the surface. The gap width appears reduced compared with the 0 h time point, and quantitative analysis of the cell-free area revealed a decrease from 923,517 pixels at 0 h to 712,306 pixels at 24 h, corresponding to a 22.9% reduction in wound area, confirming significant migratory activity. After 48 h (Figure 13c), the gap is almost completely closed. The wound area decreased to 333,095 pixels, corresponding to a 63.9% reduction relative to 0 h, demonstrating robust and progressive closure. These findings confirm the high proliferative and migratory capacity of B16-F10 cells under control conditions (DMEM), consistent with their well-established aggressive phenotype and frequent use in studies of tumor progression and metastasis [37,38].

**Figure 14:**
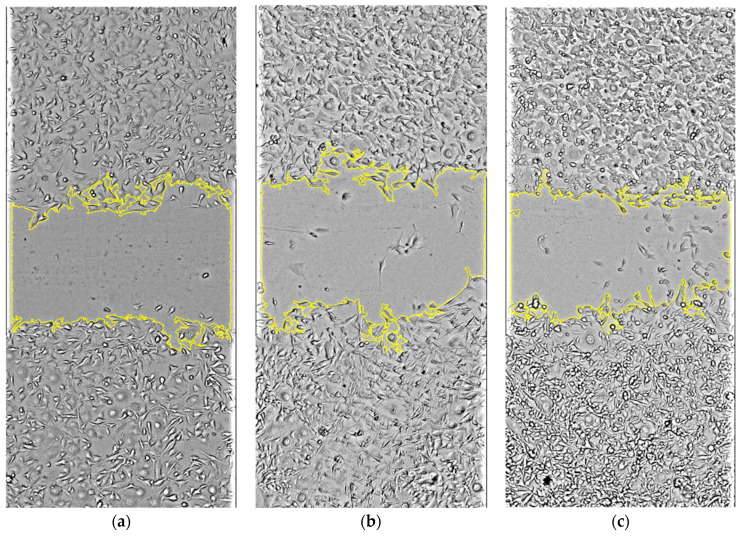
Scratch assay of murine melanoma cells (B16-F10 cell line) exposed to NFs-Chl at the IC_50_ concentration determined by the MTT assays (313.4 μM): (**a**) B16-F10 at 0 h; (**b**) B16-F10 at 24 h; (**c**) B16-F10 at 48 h.

In contrast, cells exposed to NFs-Chl at the concentration corresponding to the IC_50_ previously determined by the MTT assay (313.4 µM) exhibited evident alterations in the dynamics of scratch closure, as shown in Figure 14.

Although the initial morphology of the cellular monolayer at 0 h (Figure 14a) was similar to that observed in the control group, the progression of cell migration over time was markedly reduced. After 24 h of incubation (Figure 14b), the wound area decreased from 1,115,973 pixels to 1,027,445 pixels, corresponding to only a 7.9% reduction, indicating a strong inhibition of early migratory activity compared to the control group. In addition, an increased frequency of cells with rounded or partially retracted morphology was observed near the edges of the monolayer, a feature often associated with alterations in cell adhesion or cellular stress induced by bioactive compounds such as NFs-Chl.

After 48 h of incubation (Figure 14c), although some advancement of cells into the wound area was observed, closure remained incomplete. The wound area was reduced to 716,037 pixels, corresponding to a 39.4% decrease relative to 0 h, which is substantially lower than the closure observed in the control group (63.9%). The cellular density within the scratch area remained visibly reduced, reinforcing the inhibitory effect on migration promoted by NFs-Chl associated with PDT. This pattern suggests that exposure to NFs-Chl combined with PDT at the IC_50_ concentration negatively interferes with processes involved in cell migration, possibly by affecting cytoskeletal dynamics, cell adhesion, or signaling pathways associated with cellular motility [39,40].

Overall, the results of the scratch assay demonstrate that while B16-F10 cells in the control group exhibit progressive and efficient wound closure, exposure to NFs-Chl significantly impairs this process. The reduction in wound closure was approximately 2.9-fold lower at 24 h (22.9% vs. 7.9%) and remained markedly reduced at 48 h (63.9% vs. 39.4%), indicating a sustained inhibitory effect on cell migration. These findings are consistent with recent studies showing that compounds with antitumor potential frequently reduce cell migration in melanoma models evaluated by wound-healing assays, reflecting possible interference with cellular mechanisms associated with tumor invasion and dissemination [38].

Taken together, these preliminary in vitro results indicate that chlorophyll-containing nanoformulations exert an inhibitory effect on the migratory capacity of B16-F10 cells when associated with PDT, suggesting potential applications of these formulations as modulators of processes related to tumor progression. Nevertheless, additional in vivo studies are required to further visualize and validate this therapeutic potential.

Additionally, we emphasize that although B16-F10 and HaCaT cells originate from different species, this pairing was intentionally adopted as an initial proof-of-concept screening strategy rather than as a species-matched selectivity assay. In this context, the scratch assay provided a functional readout of the anti-migratory effect of PDT-activated NFs-Chl in B16-F10 cells, complementing the viability and proliferation data obtained from the MTT and RealTime-Glo™ assays. Thus, the present experimental design was conceived as an early-stage translational screening approach to establish preliminary evidence of photodynamic efficacy and relative cutaneous safety, providing initial indications of cellular selectivity. Nevertheless, future studies employing human melanoma cell lines and in vivo tumor models will be necessary to further validate the therapeutic relevance and translational applicability of these findings.

## 3. Materials and Methods

### 3.1. Materials and Reagents

Trypan blue, sodium bicarbonate, Dulbecco’s Modified Eagle Medium (DMEM), the surfactant Cremophor ELP^®^, and castor oil were purchased from Sigma-Aldrich, Cajamar, São Paulo, Brazil. Chlorophyll in oil was obtained from Etnobotanica, Itamonte, Minas Gerais, Brazil. Penicillin and streptomycin, fetal bovine serum (FBS), and trypsin–EDTA were purchased from Gibco, Waltham, MA, USA. The reagent 3-(4,5-dimethylthiazol-2-yl)-2,5-diphenyltetrazolium bromide (MTT) was obtained from Invitrogen. All other materials and reagents used in this study were of analytical grade and/or suitable for cell culture applications and were supplied by the Nanobiotechnology Laboratory of the Department of Genetics and Morphology at the Institute of Biological Sciences of the University of Brasília, Darcy Ribeiro Campus.

### 3.2. Preparation of Chlorophyll Nanoformulations

For the preparation of the NFs-Chl, the spontaneous emulsification method was employed, a low-energy technique that enables scalability for industrial applications [16]. Initially, Cremophor ELP^®^ (4.5 g) and castor oil (1.5 g) were mixed under magnetic stirring at 350 rpm and a temperature of 35 °C for approximately 5 min. Subsequently, while the mixture remained under magnetic stirring, chlorophyll in oil was dispersed into the surfactant/oil (S/O) mixture at different concentrations (168 µM, 420 µM, 840.5 µM, 1260 µM, and 1680 µM), prepared from a stock solution of 5000 µM. The agitation was maintained for an additional 15 min to promote interaction among the components.

Subsequently, while maintaining continuous stirring, 50 mL of phosphate-buffered saline (PBS) was added, and the system was kept under agitation for an additional 20 min, resulting in NFs-Chl with a translucent appearance. The chlorophyll-free nanoemulsions were prepared using the same method, except for the absence of the chlorophyll incorporation step in the formulation.

### 3.3. Physicochemical Characterization by Dynamic Light Scattering (DLS)

The colloidal characteristics of the NFs-Chl, including hydrodynamic diameter (Dh) and polydispersity index (PdI), were evaluated using dynamic light scattering (DLS) with a ZetaSizer Nano ZS^®^ instrument (Malvern Instruments, Malvern, UK). The zeta potential (ZP) of the nanodroplets was determined by electrophoretic mobility measurements using laser Doppler electrophoresis (LDE), performed with the same instrument (ZetaSizer Nano ZS^®^). The stability of the NFs-Chl was also assessed over a period of 365 days by monitoring these physicochemical parameters under two storage conditions: one at 25 °C and another between 4 and 8 °C. All measurements were performed at 25 °C with a detection angle of 90°. Prior to analysis, the samples were diluted with water at a ratio of 1:10 (*v*:*v*).

### 3.4. Optical Characterization by UV–Vis and Fluorescence Spectroscopy

To identify the molecular absorption bands of NFs-Chl, the methodology was based on the study by Altamimi et al. [41], with minor modifications. The analyses were performed using a Cary 50 UV–Vis molecular absorption spectrophotometer (Varian, Palo Alto, CA, USA) equipped with a pulsed xenon lamp, a 0.25 m Czerny–Turner monochromator, and a silicon diode detector. Spectral scanning was carried out in the range of 300–700 nm using a cuvette with two polished faces and an optical path length of 10 mm. All measurements were performed in triplicate at a temperature of 25 °C. In addition, a 30-day monitoring period was conducted to evaluate the stability of the optical absorbance properties of the NFs-Chl.

To obtain the fluorescence emission spectra of the NFs-Chl, an LS55 fluorometer from PerkinElmer was used. The samples were: NFs-Chl, chlorophyll-free nanoemulsion, chlorophyll solution diluted in ethanol, and chlorophyll dispersed in water, which were excited at the maximum absorbance peak (420 nm) identified in the UV–Vis spectroscopy analyses. In addition, a 30-day monitoring period was conducted to evaluate the stability of the fluorescence optical properties of the NFs-Chl stored at 25 °C.

### 3.5. Fourier Transform Infrared Spectroscopy (FTIR)

To identify the functional chemical groups of the NFs-Chl and their individual constituents, Fourier transform infrared spectroscopy (FTIR) was employed using a spectrometer (Vertex-70, Bruker Optik, Ettlingen, Germany). The analyses were performed using the attenuated total reflectance (ATR) method, with a spectral resolution of 4 cm^−1^ and 96 scans in the range of 400–4000 cm^−1^. Six samples were evaluated, namely: a physical mixture of the components (Cremophor ELP^®^, castor oil, and chlorophyll), the isolated components Cremophor ELP^®^, castor oil, and chlorophyll in oil, as well as the NFs-Chl and the chlorophyll-free nanoemulsion.

### 3.6. Raman Spectroscopy Analysis

In addition to the FTIR/ATR analyses, Raman spectroscopy was also performed using a LabRam HR Evolution spectrometer (Horiba, Paris, France), with a spectral resolution of 1 cm^−1^ in the range of 150–1800 cm^−1^. The samples were excited at a wavelength of 405 nm with a laser power of 10 mW. Three samples were evaluated: a physical mixture of the components (Cremophor ELP^®^, castor oil, and chlorophyll), the NFs-Chl, and the chlorophyll-free nanoemulsion.

### 3.7. Photodynamic Generation of Reactive Oxygen Species (ROS)

The production of ROS from the NFs-Chl was evaluated using the degradation method of 1,4-diphenyl-2,3-benzofuran (DPBF) when exposed to singlet oxygen (^1^O_2_), based on the study by Muehlmann et al. [16], with minor modifications. The DPBF solution was prepared at a concentration of 0.22 mg/mL in absolute ethanol and subjected to an ultrasonic bath for 10 min until a homogeneous solution was obtained. Subsequently, 20 µL of the DPBF solution and 180 µL of each sample were added to the wells of a 96-well microplate. The samples evaluated included NFs-Chl, chlorophyll-free nanoemulsion, chlorophyll solution, and chlorophyll dispersed in water.

The microplates were then exposed to LED irradiation at a wavelength of 660 nm (108 J/cm^2^) at a temperature of 25 °C, with a distance of 10 cm between the light source and the plate. Each irradiation cycle lasted 10 s per scan, resulting in a total of 30 scans. After each irradiation cycle (10 s), the microplates were read at a wavelength of 410 nm using a SpectraMax M2 spectrophotometer (Molecular Devices, San Jose, CA, USA), followed by another 10 s LED exposure and subsequent spectrophotometric reading. This process was repeated until all 30 scans were completed. All experiments were performed in triplicate.

### 3.8. Morphological Analysis by Transmission Electron Microscopy (TEM)

For morphological analysis of the NFs-Chl (1500 µM), transmission electron microscopy (TEM) was performed using a JEOL 1011 microscope (Tokyo, Japan). Briefly, the samples were previously diluted at a ratio of 1:10 (*v*:*v*) with distilled water, and, subsequently, 5 µL of the diluted sample was deposited onto a copper grid coated with a Formvar film, which was left to dry for 24 h. After drying, the samples were contrasted for 3 min in 2% osmium tetroxide vapor prior to TEM analysis. For observation, the instrument was operated at an accelerating voltage of 80 kV and images were acquired at a magnification of 20,000× for morphological evaluation of the NFs-Chl and determination of their equivalent circular diameters.

### 3.9. Biological Assays

#### 3.9.1. Cell Culture Conditions

The murine melanoma cell line B16-F10 and the spontaneously immortalized human keratinocyte cell line HaCaT were obtained from the Banco de Células do Rio de Janeiro (BCRJ, Rio de Janeiro, RJ, Brazil). The culture of murine melanoma cells (B16-F10) and keratinocytes was performed in Dulbecco’s Modified Eagle Medium (DMEM) supplemented with 10% fetal bovine serum (FBS) and antibiotics (penicillin (100 µg/mL) and streptomycin (100 µg/mL) at a temperature of 37 °C in a cell incubator with 5% CO_2_.

#### 3.9.2. Photodynamic Therapy Treatment

The photodynamic evaluation of NFs-Chl was performed based on the protocol described by Morais et al. [42], with minor modifications. Initially, murine melanoma cells (B16-F10) were seeded in 96-well microplates at a density of 1 × 10^4^ cells per well. Subsequently, the cells were exposed to NFs-Chl, chlorophyll-free nanoemulsions, and chlorophyll solutions at different concentrations (93.75, 187.5, 375, 750, and 1500 µM, respectively) for 30 min. After incubation, the samples were discarded and the cells were washed twice with phosphate-buffered saline (PBS). The cells were then exposed to light-emitting diode (LED) irradiation (660 nm, 108 J/cm^2^) for 15 min. Following LED exposure, the cells were maintained in the dark for 24 h in a cell incubator containing 5% CO_2_. The same protocol was applied to human keratinocytes (HaCaT).

#### 3.9.3. Cell Viability Assays by MTT

Cell viability experiments were performed using the 3-(4,5-Dimethylthiazol-2-yl)-2,5-diphenyltetrazolium bromide (MTT) assay. Briefly, after 24 h of treatment with photodynamic therapy (PDT), 96-well microplates containing 1 × 10^4^ murine melanoma cells (B16-F10) per well received MTT at a final concentration of 0.5 mg/mL. The microplates were then incubated for 2 h at 37 °C in a cell incubator with 5% CO_2_.

Subsequently, the supernatant was removed, and the dark blue formazan crystals formed were dissolved by adding 100 µL of DMSO. The microplates were read using a SpectraMax M2 spectrophotometer (Molecular Devices, San Jose, CA, USA) at an absorbance of 595 nm, corresponding to the characteristic absorbance wavelength of MTT. The results were expressed as a percentage relative to the control group (cells exposed only to DMEM). Each treatment was performed in triplicate and repeated in three independent experiments. The same protocol was applied to human keratinocytes (HaCaT).

#### 3.9.4. Cell Survival Calculation

The determination of the survival fraction of murine melanoma cells (B16-F10) and human keratinocytes (HaCaT) was based on an adaptation of the method described by Kim et al. [43] and later applied in studies by Nikzad et al. [29]. Briefly, the analysis of the results obtained from the MTT assays revealed a linear correlation between optical density values and the number of viable cells. The survival curve was constructed using a semi-logarithmic scale, representing the survival fraction as a function of concentration. The survival fraction was then determined using the following equation:

$$Survival\;Fraction\;=\frac{Mean\;OD\;in\;test\;wells\;-\;Mean\;OD\;in\;cell\;free\;wells}{Mean\;OD\;in\;control\;wells\;-\;Mean\;OD\;in\;cell\;free\;wells}$$

### 3.10. Real-Time Cell Proliferation Assays

To evaluate the long-term photodynamic effect promoted by NFs-Chl, real-time cell viability assays were performed using the B16-F10 and HaCaT cell lines with the RealTime-Glo™ MT Cell Viability Assay kit (G9711, Promega, Madison, WI, USA), following the manufacturer’s instructions. This assay is based on the detection of cell viability through luminescence generation. The pro-substrate (MT Cell Viability Substrate) diffuses into the cells, where it is reduced to form a substrate that subsequently exits the cell and reacts with the enzyme (NanoLuc^®^ Enzyme, Promega, Madison, WI, USA) present in the medium. Only metabolically active cells are capable of reducing the pro-substrate; therefore, luminescence production is proportional to the number of viable cells. This luminescence can be continuously monitored, allowing assessment of cellular health without interrupting the experiment.

For the experiment, both cell lines were seeded at a density of 1.5 × 10^3^ cells per well in white, opaque-walled 96-well microplates (Kasvi, model K12-096, Pinhais, Paraná, Brazil). After 24 h of incubation to allow cell adhesion to the bottom of the wells, the cells were treated with 100 µL of DMEM containing NFs-Chl, NFs-free, or Sol-Chl previously diluted in complete culture medium at the IC_50_ concentration determined from the MTT assays (313.4 µM). Subsequently, the MT Cell Viability Substrate and the modified luciferase (NanoLuc Enzyme) were equilibrated at 37 °C and diluted in cell culture medium in the following proportion: 996 µL of medium, 2 µL of substrate, and 2 µL of enzyme. The solution was homogenized, and 100 µL of the reagent mixture was added to each well immediately after exposure to PDT treatment, as described in Section 3.9.2.

The plates were gently mixed and incubated, protected from light. Luminescence intensity (RLU) was measured from 1 h to 48 h after reagent addition using a microplate reader (Varioskan™ LUX, Thermo Fisher Scientific, Massachusetts, USA). The results were expressed as mean ± standard deviation (SD) from triplicate determinations obtained from three independent experiments.

### 3.11. Scratch Assay

The migratory capacity of the B16-F10 and HaCaT cell lines was evaluated using the scratch assay, as described in studies by Moreira et al. [44]. This method consists of creating a wound-like gap in a confluent cell monolayer and monitoring the closure of this area over time. This approach allows assessment of whether cell migration is reduced following exposure of cells to NFs-Chl at the IC_50_ concentration determined from the MTT assays (313.4 µM), in comparison with the control group (DMEM).

For this assay, both cell lines were seeded in 12-well plates at a density of 1.5 × 10^5^ cells per well and cultured until a confluent monolayer was formed. After removal of the culture medium, a straight scratch was manually created across the monolayer using a 200 µL plastic pipette tip. Subsequently, the wells were washed once with 1 mL of PBS and exposed to treatment with NFs-Chl (313.4 µM) or to the control condition containing only culture medium (DMEM). Representative images of the cell proliferation and migration process were recorded using phase-contrast microscopy with the automated cell imaging system Invitrogen EVOS FL (Thermo Fisher Scientific, USA) immediately after wound creation and treatment exposure to define the initial wound area. Additional images were acquired after 24 h and 48 h.

The images were analyzed using the ImageJ software Version 1.54p 17 February 2025. The assay was performed with technical triplicates for each condition (*n* = 3). The percentage reduction in the wound area was calculated using the formula described by Moreira et al. [44]:

$$Percentage\;of\;scratch\;area\;closure\;=\;100\%\;-\left(\frac{Final\;area}{Initial\;area}\right)\;\times \;100$$

### 3.12. Statistical Analysis

Biological data were analyzed using GraphPad Prism software version 9.5.0 (GraphPad Software, La Jolla, CA, USA). Results are expressed as mean ± standard deviation (SD) from three independent experiments (*n* = 3). Statistical analyses were performed using two-way repeated-measures analysis of variance (two-way RM ANOVA), with treatment and concentration or time considered as independent factors, as appropriate for each experiment. When the assumption of sphericity was violated, the Geisser–Greenhouse correction was applied. Statistical significance was defined as *p* < 0.05.

## 4. Conclusions

The results presented herein demonstrate that chlorophyll-containing nanoformulations (NFs-Chl), when combined with photodynamic therapy, promote a significant reduction in metabolic activity and proliferation of the murine melanoma B16-F10 cell line compared with chlorophyll solution in ethanol (Sol-Chl) and chlorophyll-free nanoformulations (NFs-free). The temporal viability curve revealed that NFs-Chl maintained metabolic signals close to background levels over 48 h, whereas Sol-Chl exhibited a loss of photodynamic activity over time (from approximately 15–18 h). In addition, NFs-Chl markedly reduced cell migration in the scratch assay, evidencing an inhibitory effect on melanoma cell migratory behavior under the in vitro experimental conditions evaluated.

These findings indicate that NFs-Chl represent a promising alternative to enhance the delivery and efficacy of natural photosensitizers in PDT against melanoma under preliminary in vitro conditions. In this context, future studies should focus on: (i) investigating the molecular mechanisms underlying the observed photodynamic cytotoxicity; and (ii) evaluating biodistribution, toxicity, and therapeutic efficacy in relevant in vivo tumor models. These steps are essential to generate robust data required for advancing toward clinical studies and for supporting future translational investigations.

## Figures and Tables

**Table 1:** Physicochemical characterization of chlorophyll nanoformulations (NFs-Chl) and chlorophyll-free nanoemulsion determined by dynamic light scattering (DLS).

Samples	HD (nm)	PdI	ZP (mV)	pH
NFs-Chl (168 μM)	24.17 ± 0.56	0.056 ± 0.009	0.43 ± 0.91	6.33
NFs-Chl (420 μM)	27.14 ± 0.10	0.107 ± 0.005	−1.21 ± 1.20	6.32
NFs-Chl (840.5 μM)	28.38 ± 0.17	0.120 ± 0.006	−0.73 ± 1.14	6.33
NFs-Chl (1.26 × 10^3^ μM)	30.25 ± 0.65	0.099 ± 0.656	−1.84 ± 0.46	6.32
NFs-Chl (1.681 × 10^3^ μM)	30.71 ± 0.11	0.126 ± 0.008	−1.84 ± 0.85	6.31
Chlorophyll-free nanoemulsion	25.28 ± 0.36	0.096 ± 0.009	−2.26 ± 1.73	6.34

Note: data are expressed as mean ± standard deviation. Abbreviations: NFs-Chl, chlorophyll nanoformulation; HD, hydrodynamic diameter; nm, nanometers; PdI, polydispersity index; ZP, zeta potential.

## Data Availability

The original contributions presented in this study are included in the article. Further inquiries can be directed to the corresponding authors.

## Abbreviations

The following abbreviations are used in this manuscript:

DBB	1,3-dibenzoylbenzene
DPBF	1,4-diphenyl-2,3-benzofuran
FTIR	Fourier-Transform Infrared
LDE	Laser Doppler Electrophoresis
PdI	Polydispersity index
HD	Hydrodynamic Diameter
ZP	Zeta Potential
O_2_	Molecular Oxygen
^1^O_2_	Singlet Oxygen
ROS	Reactive Oxygen Species
Chl	Chlorophyll
PDT	Photodynamic Therapy
TEM	Transmission Electron Microscopy
FBS	Fetal Bovine Serum
DMEM	Dulbecco’s Modified Eagle Medium
MTT	Reagent 3-(4,5-dimethylthiazol-2-yl)-2,5-diphenyltetrazolium bromide
PBS	Phosphate-Buffered Saline
S/O	Surfactant/Oil
nm	Nanometer
µM	Micromolar
IC_50_	Maximum Inhibitory Concentration of 50%
NFs-Chl	Chlorophyll-loaded Nanoformulations
Sol-Chl	Chlorophyll Solution Diluted in Ethanol
Blank NFs	Chlorophyll-Free Nanoemulsion
